# Adverse Conditions, Psychological Aspects, and Teachers’ Tendency Toward Sustainability and a Less Conservative Future

**DOI:** 10.3389/fpsyg.2022.843258

**Published:** 2022-07-11

**Authors:** Ilaria Di Maggio, Maria Cristina Ginevra, Sara Santilli, Laura Nota

**Affiliations:** Department of Philosophy, Sociology, Education and Applied Psychology, University of Padova, Padua, Italy

**Keywords:** teachers, COVID-19, state anxiety, personal need for structure, propensity toward a more conservative socio-economic vision, propensity toward sustainability

## Abstract

This study aimed to examine the effect of cognitive priming linked to the COVID-19 pandemic, through state anxiety and personal need for structure, on teachers’ tendency toward sustainability and teachers’ tendency toward a conservative socio-economic vision. We involved a sample of 984 Italian teachers, and by manipulating the saliency of the COVID-19 pandemic, we found that the saliency of the COVID-19 pandemic positively impacted state anxiety and that state anxiety impacted teachers’ tendency toward sustainability both directly and indirectly through the mediational role of the personal need for structure. Finally, we found that state anxiety only indirectly through the personal need for structure impacted teachers’ tendency toward a conservative socio-economic vision.

## Introduction

Although different areas of the world have been challenged over the centuries by several epidemics and pandemics, the entire planet has recently been alarmed and challenged by an extensive, unprecedented, troublesome epidemic outbreak: COVID-19 pandemic. It is seen as a new kind of particularly contagious coronavirus with disastrous effects not only on the health and psychological wellness of individuals but also on society in general (Rossi et al., 2020; Wang et al., 2020). The COVID-19 pandemic was first reported in Wuhan, Hubei province, China, in late 2019. On 11 March 2020, due to the exponential increase in the cases of infection all over the world, COVID-19 was declared a global pandemic by the World Health Organization.

The impacts of the COVID-19 pandemic on the health and wellbeing of the world population have immediately attracted the interest of numerous researchers. Specifically, several studies have found that quarantine measures, as well as the COVID-19 pandemic itself, were associated with anxiety, depression, stress, and posttraumatic stress symptoms (Gan et al., 2020; Pandey et al., 2020; Rossi et al., 2020; Wang et al., 2020). This is in line with the effects of negative consequences of epidemics and pandemics that have affected the world in recent years (Brooks et al., 2020).

However, few studies have investigated the effects of cognitive priming linked to the COVID-19 pandemic on the behavior of individuals toward the construction of a more inclusive, ethical-social, and sustainable society, specifically in career issues. In particular, with the term “sustainability,” we refer to the concept of sustainable development that “meets the needs of the present without compromising the ability of future generations to meet their own needs” (UN World Commission on Environment and Development [WCDE], 1987, p. 43). It is an integrative concept involving environmental, social, and economic aspects in order to create diverse, healthy, and solidary societies that meet the needs of the present without compromising the ability of future generations to meet their own needs (UCLA Sustainability Committee., 2016).

The importance to analyze contextual features in career guidance has been emphasized by the career construction adaptation model (Savickas et al., 2009; Savickas and Porfeli, 2012), which considers career development as a result of the combination of individual and environmental features. It emphasizes the ability to adapt to the context, underlining the need to develop knowledge and skills to examine non-linear causalities, complex dynamics, ecological settings, and multiple subjective contexts. Recently, different career scholars—within the career construction adaptation model—have emphasized the relevance to take into account the new global challenges and the need to consider career development from the perspective of inclusion and sustainability (Guichard, 2018; Nota et al., 2020).

The few studies that focused on this topic often achieved different results. On the one hand, research on the general adult population showed how the pandemic increased the propensity to consider issues related to sustainable development both in consumer choices (Hörisch, 2021) and in some daily life behaviors (Severo et al., 2021). On the other hand, further studies investigated the effect of the pandemic in career contexts. For example, Pressley (2021) involved teachers of different origins and degrees and found that the pandemic, together with the anxiety and workload associated with it, led the participants to drift away from a positive tendency toward inclusion. The achieved outcomes, even if incoherent at a first glance, are in line with what had been stated by Karwowski et al. (2020). In their opinion, the behavior of different individuals during a pandemic is not directly connected to defense strategies against pathogens, but they are connected to the state of perceived anxiety and the personal need for structure. In addition, Di Maggio (2022), inspired by the study by Karwowski et al. (2020) and involving 540 Italian teachers, showed how the perceived state of anxiety rises because of cognitive priming linked to the COVID-19 pandemic instead of behaving as it would when compared to a neutral cognitive prime. This rise in perceived state anxiety was associated with a more negative attitude toward inclusion.

Based on these premises, this study aimed at examining for the first time not only the mediational role of state anxiety but also the personal need for structure in the relationship between cognitive priming—a stimulus able to temporarily increase the accessibility of some thoughts and ideas (Berkowitz, 1984)—linked to the COVID-19 pandemic and the adherence to a functional tendency for the construction of more sustainable and inclusive societies in their career activities. In this study, we focused on teachers as they play an important role in the process of building the knowledge, skills, awareness, values, and sustainable actions necessary to achieve the goal of an inclusive and sustainable planet in the new generation (Kalu and Ali, 2004; Borges, 2019; Nousheen et al., 2020). Based on the results obtained by Karwowski et al. (2020), we hypothesized that the personal need for structure could have an additive effect in increasing adherence to the less inclusive and sustainable tendencies in teachers’ career activities. Said actions, as already noticed, depend on the level of experienced anxiety (Di Maggio, 2022). The aforementioned need for structure may also lead teachers to inadvertently share these more conservative tendencies through their career activities.

## Teachers’ Tendency Toward Sustainability and Less Conservative Future

In the area of career guidance and career counseling, scholars have recently been focusing their attention toward social challenges through personal and career development and actions (Kenny et al., 2019; Di Maggio et al., 2020), supporting, in this way, a sustainable and inclusive development. This idea refers to the protection of the global planet, focusing on the social, economic, and environmental points of view, considering them as analogous and mutually dependent. This concept should guide people in their everyday choices. The goal is to improve global, social, and personal wellbeing and to promote a more informed and livelier “presence in the world,” decent jobs, and education (Kargulowa, 2018). Consequently, people should be stimulated to be less “egocentric,” to identify barriers related to inequalities, discriminations, and exploitations also even during their own career activities. Individuals should be encouraged to choose occupational experiences that enable them to reach wellness and, to being motivated by less individualistic values to support the creation of inclusive and sustainable social contexts (García-Feijoo et al., 2020; Nota et al., 2020; Santilli et al., 2020).

Based on these premises, teachers’ consideration of the challenges of sustainable development in career activities is a critical factor to promote in their students’ positive tendency toward inclusion and sustainability (Kalu and Ali, 2004; Borges, 2019; Nousheen et al., 2020). As a matter of fact, education and the achievements of students largely depend on the teacher’s effectiveness (Laurie et al., 2016; Darling-Hammond, 2017; Kearney and Garfield, 2019). In this regard, for example, Ojala and Bengtsson (2019) found that young people who have the capability of coping in this more constructive way are more inclined to use coping strategies and become active democratic citizens if they perceive that their teachers greatly emphasize social problems and whether they share responsibility for an equitable and sustainable future with their students. This is in line with Lombardi and Sinatra (2013) study founding that teachers’ emotions and attitudes concerning topics such as inclusion and sustainability in their career activities have an impact on their communication with their students and the development of their positive tendency toward inclusion and sustainability.

The research that analyzed the tendency toward sustainability in career activities reached conflicting results. Some underlined that teachers have a positive attitude toward an inclusive and sustainable education (Kalu and Ali, 2004; Todorovic et al., 2011), and others report that teachers show a moderate positive tendency toward an inclusive and sustainable education (Avramidis et al., 2000; Alghazo et al., 2003; Keles, 2017). On the contrary, there seem to be few studies in the literature that evaluate the impact of the COVID-19 pandemic on teachers’ tendency toward sustainability in their career activities. The same is true for the propensity toward a more environmental and socially oriented economy. As a matter of fact, the research, as highlighted in the previous lines, has shown that the pandemic and the anxiety associated with it are connected to a less positive attitude toward inclusion (Pressley, 2021; Di Maggio, 2022). Similarly, the study by Karwowski et al. (2020) found that the pandemic, affecting state anxiety and the personal need for structure, has influenced attitudes and the propensity toward conservatism, that is to say, the propensity to give attention to a more traditional and well-known idea of socioeconomic reality based on a competitive market.

## State Anxiety and Personal Need for Structure as Mediators Between the COVID-19 Pandemic and Teachers’ Tendency Toward Sustainability and a Less Conservative Future

Different studies showed as state anxiety symptoms feelings of tension, apprehension, nervousness, and worry (Spielberger and Rickman, 1990), which were associated with several COVID-19 pandemic stressor factors, such as health status of relatives (Flesia et al., 2020; Maaravi and Heller, 2020), increased use of preventive measures (Wong et al., 2020), being under quarantine, the interruption of work, and having experienced a stressful life event (Rossi et al., 2020). Moreover, Di Blasi et al. (2021) showed that, despite different pandemic periods having quantitatively and qualitatively different impacts on the psychological health of individuals, state anxiety symptoms, unlike depressive symptoms or fear of COVID, seem to remain constant during both lockdown and reopened periods. Di Blasi et al. (2021) explained these results by referring to the uncertainty with respect to what will happen that remains constant over both lockdown and reopened periods.

As regards teachers and state anxiety, some studies carried out during the COVID-19 pandemic have found a high prevalence of state anxiety among teachers, varying from 10 to 49.4% (Silva et al., 2021). For instance, Allen et al. (2020) detected high anxiety levels in teachers’ when schools were closed and news reported information about their reopening. Similar results were identified by Pressley (2021): During the pandemic, teachers suffered high levels of anxiety connected to the pandemic, teaching requests, and interactions with parents. In addition, anxiety, dissimilarly from other variables such as location, instruction type, years of teaching experience, and ethnicity was useful to predict teachers’ burnout.

Even if state anxiety *per se* is intended as a normal reaction/response to a stressful or threatening circumstance that supports people’s adaptation to the environment, when sustained it can interfere with the activities of daily life, becoming maladaptive for the individual (Schmidt, 2020). A potential negative effect of elevated state anxiety, which can also become maladaptive, is connected to an increase in personal need for structure (Sollár, 2008), that is to say, a desire for predictability experienced by individuals during pandemic periods (Neuberg and Newsom, 1993).

A distinction between *state* and *trait* personal need for structure is needed (Sollár, 2008). Whereas *state* personal need for structure refers to situationally evoked and activated in urgent and ambiguous conditions (Kruglanski and Freund, 1983), *trait* personal need regards a typical way of individuals’ reacting which will be evident regardless of the character of the situation (Thompson et al., 2013). In this study, we focused on *state* personal need for structure.

Sollár (2008), considering the *state* personal need for structure, found a positive correlation between this construct and the state of anxiety. Moreover, Karwowski et al. (2020) showed that the COVID-19 pandemic impacts the personal need for structure through the mediational role of state anxiety. Lastly, studies have observed that *trait* or *state* personal need for structure is positively associated with a less propensity to change own attitudes and stereotypes (e.g., Freund et al., 1985; Thompson et al., 2013; Karwowski et al., 2020).

## Aim of the Study

This study addressed the gap in the literature about the lack of studies that analyze the mediational role of state anxiety and personal need for structure during the COVID-19 pandemic and variables connected to sustainability and less conservative future for teachers. This study aimed at assessing, for the first time, the impact of the COVID-19 pandemic on state anxiety, the personal need for structure, teachers’ tendency toward sustainability, and teachers’ tendency toward a conservative socio-economic vision. It was expected that the exposure to cognitive priming linked to the COVID-19 pandemic would have increased teachers’ state anxiety levels and personal need for a structure that, in turn, would have been associated with a lower propensity to consider the challenges related to sustainable development (tendency toward sustainability) and a greater propensity toward a more conservative socio-economic vision (tendency toward conservative socio-economic vision). Specifically, based on Karwowski et al. (2020) and Di Maggio (2022), we hypothesized that state anxiety and the personal need for a structure mediating the relationship between cognitive priming linked to the COVID-19 pandemic and teachers’ tendency toward sustainability and toward a conservative socio-economic vision.

## Materials and Methods

### Experimental Design

To test the hypotheses of the study, an experimental design was developed based on Karwowski et al. (2020) study. Specifically, Karwowski et al. (2020) manipulated the saliency of the COVID-19 pandemic using different press media reports: neutral texts (e.g., brief texts with information about foldable smartphones) and a text about the COVID-19 pandemic (see https://osf.io/f9pgq to access the original stimuli used by the authors). Similarly, to manipulate the saliency of the COVID-19 pandemic, we presented to participants, in the first step of the study, a brief draft with information about the COVID-19 pandemic in the experimental condition and a brief draft with information about the foldable smartphone in the control condition. Moreover, participants in both groups were asked to answer a multiple-choice question aimed at investigating text comprehension (The Italian language stimuli used in this study are presented in Appendix 1, and there is also an English version of the stimuli to facilitate the interpretation for international readers). Following, all participants in both conditions (experimental and control) were presented with the measures aimed to assess the dependent variables of the study.

### Participants

Sample size was calculated using G* Power 3 (Faul et al., 2007). A total sample of 895 participants was needed considering a small effect size and a power of 0.95. Following Schwedes and Wentura (2016) study, as more outcomes were used, we decided to increase the sample size. A sample of 984 northern Italian teachers (106 men and 878 women) with a mean age of 39.52 years, ranging from 21 to 67 years (SD = 39.52), was involved in this study. Half of the sample, controlling for gender, was randomly assigned to the experimental condition (492 participants) and the other half to the control condition (492 participants). No age-related differences between the two groups were found [*t*(982) = −0.270; *p* = 0.787].

### Measures

#### State Anxiety

State anxiety was tested using the Italian version of the Positive and Negative Affect Scale (Terracciano et al., 2003). PNAS consists of 20 items assessing positive and negative mood, and it is considered as a measure to assess mood related to anxiety (Schalet et al., 2014). Participants rated the extent to which they have experienced at a particular time frame (in this study “at this moment”) the mood investigated by the single items using a five-point rating scale ranging from 1 (*very slightly or not at all*) to 5 (*extremely*). Following Karwowski et al. (2020) study, the state anxiety was assessed considering only the items referring to a mood related to anxiety (“Nervous,” “Jittery,” “Scared,” and “Distressed”). Cronbach’s α internal-consistency reliability for this study was 0.88.

#### Personal Need for Structure

Following Karwowski et al. (2020) study, the personal need for structure was tested using the Italian version of the personal need for structure scale (PNS; Vannucci et al., 2011). PNS (Neuberg and Newsom, 1993) is the most commonly used measure of individual differences in the desire for structure (Aldi et al., 2020) and consists of 12 items (e.g., “I enjoy having a clear and structured mode of life”). Participants were asked to read each item and decide how much they agree with each according to their tendencies, beliefs, and experiences considering a 6-point scale (1 = *strongly disagree* and 6 = *strongly agree*). The total score ranged from 12 to 72; higher scores indicate a higher desire for structure, a stronger preference for certainty, and a dislike for ambiguity. The PNS showed adequate reliability (Cronbach’s alpha = 0.77), a test–retest reliability of 0.76, and good convergent and divergent validity (Neuberg and Newsom, 1993). The Italian version showed psychometric properties consistent with the English version (Vannucci et al., 2011). Cronbach’s α internal-consistency reliability for this study was 0.80.

#### Tendency Toward a Conservative Socio-Economic Vision in Career Activities

To test the tendency toward conservative socio-economic vision in teachers’ career activities, we used a subscale of the self-report measures “Thoughts about future development and economy in career field” developed by Nota et al. (2020). Specifically, this measure is a 11-item scale to analyze thoughts with a conservative vision of economics (five items, e.g., “*In order to promote employment and personal fulfillment, we should give more importance to competition because it encourages people to put more effort into their actions and develop new ideas.”*) and thoughts with a more equitable and supportive conception of it (six items, e.g., “*People need to take action in order to reduce poverty and unemployment. If they do not struggle to look for a job by themselves, they will never find it.”*). Participants responded to each item on a scale ranging from 1 (*you judge that thought extremely unsuitable)* to 5 (*you judge that thought completely suitable*). As reported by Nota et al. (2020), the questionnaire is a psychometrically valid and reliable measure. In this study, we used only the subscale composed of five items for assessed thoughts with a conservative vision of economics. Cronbach’s α internal-consistency reliability for this factor was 0.68.

#### Tendency Toward Sustainability in Career Activities

To test the tendency toward sustainability in teachers’ career activities, we used the self-report measure “The future is around the corner. What will it hold for us? An instrument on UN’s goals for the inclusive and sustainable development” developed by Nota et al. (2020). Specifically, this 17-item questionnaire refers to the 17 goals presented in the 2030 Agenda for Sustainable Development. Each participant was asked how much he/she thinks that every goal presented can affect his/her educational and career choices/activities. An example of an item is the following: “In the future, there will certainly be much to do to ensure employment and decent work for all… How could this topic influence your educational and career choices/activities?” Participants were invited to express their views on a five-point Likert scale (1 = very little, 5 = very much). Nota et al. (2020) proved that the questionnaire is a psychometrically valid and reliable measure. Specifically, Nota et al. (2020) showed good statistics fit indices for a single factor, second-order structure of the instrument. Cronbach’s α internal-consistency reliability for this study was 0.95.

### Procedure

Through the collaboration of various educational institutions, a pool of teachers were asked by email to join this research project. The teachers were informed that participation in the study was voluntary, the survey was confidential, and they could withdraw from the survey at any time if they did not want to continue. Then, the adhesion and online informed consent was obtained, and the participants were randomly assigned, controlling for gender, to the two conditions (experimental vs. control). Later, using the free software Google Forms, an online survey was sent to participants. Specifically, in the first web page of the online survey, participants found the stimuli used for the experimental manipulation, and then, having completed the multiple-choice questions related to the proposed text, they answered the other measures. Data were collected from February to April 2021 during the third wave of the COVID-19 pandemic in Italy.

During this third wave of COVID, approximately from January to April 2021, Italian teachers were called to implement alternative teaching approaches, including socially distanced classrooms, hybrid teaching, virtual instruction, or 100% face-to-face learning. The adoption of these different teaching approaches varied constantly based on regional pandemic indices but also with respect to the specific conditions of singular classes (e.g., notification of a positive case in the classroom to be brought entirely in quarantine and therefore in virtual instruction; see Italian Decree Law n. 2 of January 14, 2021).

### Data Analysis

#### Preliminary Analysis

As suggested by Stage et al. (2004), different preliminary analyses, such as Means, standard deviations, and matric correlations, were carried out to test the assumptions in order to proceed with structural equation modeling analyses.

#### Hypothesized Model

To test the hypothesized model, we used a path analysis because an observed variable was involved in the study as an exogenous variable. Path analysis is considered an extension of the regression model designed to estimate the magnitude and significance of hypothesized causal connections among sets of exogenous and endogenous variables and to test the fit of a correlation matrix with a causal model (Stage et al., 2004). Specifically, to test the causal model goodness of fit, we used, as suggested by Smith and McMillan (2001), the following indices: the root mean square error of approximation (RMSEA), the comparative fit index (CFI), the non-normed fit index (NNFI), and the standardized root mean square residual (SRMR). Acceptable model fit was defined by the following multiple cutoff values: RMSEA ≤ 0.06, CFI ≥ 0.95, NNFI ≥ 0.95, SRMSR ≤ 0.08. Moreover, we tested the indirect effects using the product of coefficient method and generated asymmetric confidence intervals using PRODCLIN (MacKinnon et al., 2007).

## Results

### Preliminary Analysis

Means, standard deviations, and Pearson’s correlation were calculated (see Table 1). Pearson’s correlation analysis showed positive and significant correlations between the condition (control and experimental condition) and state anxiety. No significant correlations were found among the condition (experimental and control) and personal need for structure, tendency toward a conservative socio-economic vision in career activities, and tendency toward sustainability in career activities. Instead, positive and significant correlations between state anxiety and personal need for structure and negative and significant correlations between state anxiety and tendency toward sustainability in career activities were found. No significant correlations were found between state anxiety and tendency toward a conservative socio-economic vision in career activities. Moreover, Pearson’s correlation analysis showed positive and significant correlations between personal need for structure and tendency toward a conservative socio-economic vision in career activities and negative and significant correlations between personal need for structure and tendency toward sustainability in career activities. These findings emphasized the relevance of proceeding with the path analysis. Moreover, as suggested by Stage et al. (2004), we used correlation analysis to revise the model to be tested by excluding relationships between variables that in correlation were not found to be significant. Specifically, the relationship among condition (experimental and control) and personal need for structure, tendency toward a conservative socio-economic vision in career activities, and tendency toward sustainability in career activities has been excluded from the model as well as the relationship between state anxiety and tendency toward the conservative socio-economic vision in career activities.

**TABLE 1:** Means, standard deviations, and Pearson’s correlation.

Study variables	M	SD	(1)	(2)	(3)	(4)	(5)
(1) Condition (Control = 0 vs Experimental = 1)				0.192**	0.004	0.017	0.003
(2) State Anxiety	10.27	4.07			0.283**	–0.021	−0.112**
(3) Personal Need for Structure	41.98	8.75				0.104**	−0.113**
(4) Tendency toward conservative socioeconomic vision in career activities	14.94	3.53					–0.044
(5) Tendency toward sustainability in career activities	68.92	11.92					

**p < 0.01.

### Path Analysis

**FIGURE 1:**
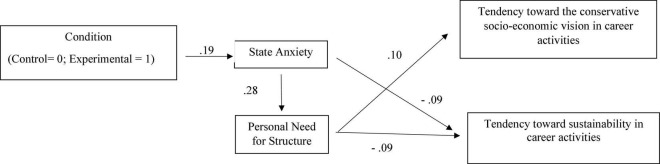
Standardized parameter estimates in the model tested. Solid lines indicate statistically significant effects.

The hypothesized causal model tested showed good fit indices: χ^2^_(5)_ = 8.089 RMSEA = 0.025; CFI = 0.978, NNFI = 0.096; SRMR = 0.021. As regards the magnitude and significance of hypothesized causal connections tested in the model (see Figure 1), we found significant and positive relationships between the saliency of COVID-19 pandemic (Condition: Control = 0 vs Experimental = 1) and state anxiety (β = 0.192; *t* = 6.14). Moreover, positive and significant relationships between state anxiety and personal need for structure (β = 0.28; *t* = 9.26) and negative and significant relationships between state anxiety and tendency toward sustainability in career activities (β = −0.087; *t* = −2.63) were found. Finally, the analysis showed a positive relationship between personal need for structure and tendency toward a conservative socio-economic vision in career activities (β = 0.104; *t* = 3.27) and negative relationships between personal need for structure and tendency toward sustainability in career activities (β = −0.088; *t* = −2.68).

Regarding the indirect effects (see Table 2), it was found that the 95% confidence intervals for the indirect effect between the saliency of the COVID-19 pandemic and personal need for structure through state anxiety ranged from 0.601 to 1.339. The 95% confidence intervals for the indirect effect between the saliency of COVID-19 pandemic and the tendency toward sustainability in career activities through state anxiety ranged from 0.101 to 0.744. The 95% confidence intervals for the indirect effect between state anxiety and tendency toward a conservative socio-economic vision in career activities through personal need for structure ranged from 0.010 to 0.043. The 95% confidence intervals for the indirect effect between state anxiety and tendency toward sustainability in career activities through personal need for structure ranged from −0.131 to −0.019. Finally, the 95% confidence intervals for the indirect effect between the saliency of the COVID-19 pandemic toward state anxiety and personal need for structure on tendency toward a conservative socio-economic vision ranged from 0.195 to 0.011 and on tendency toward sustainability toward in career activities ranged from −1.427 to −0.0275.

**TABLE 2:** Standardized direct and indirect effects of condition on tendency toward the conservative socioeconomic vision in career activities and tendency toward sustainability in career activities via state anxiety and the personal need for structure.

	State anxiety		Personal need for structure		Tendency toward the conservative socio-economic vision in career activities		Tendency toward sustainability in career activities	
	DE	IE	DE	IE	DE	IE	DE	IE
Condition	S = 0.192*							
State anxiety			S = 0.280*				S = −0.087*	
Personal need for structure					S = 0.104*		S = −0.088*	
Condition -> State anxiety				S = 0.055*				S = −0.022^b^
State anxiety -> Personal need for structure						S = 0.029^b^		S = −0.025^b^
Condition -> State anxiety-> Personal need for structure						S = 0.040^b^		S = −0.513^b^

DE, direct effect; IE, indirect effect; S, standardized. ^b^Significance of mediated effect with PRODCLIN. *Statistically significant effects with a p < 0.05.

## Discussion

This study aimed at analyzing the effect of the saliency of the COVID-19 pandemic, through state anxiety and personal need for structure, on teachers’ tendency toward sustainability and teachers’ tendency toward a conservative socio-economic vision. The preliminary analyses carried out showed, as hypothesized, the importance to test only the indirect interactions between COVID-19 pandemic, the tendency toward sustainability in career activities, and the tendency toward a conservative socio-economic vision in career activities.

Then, the path analysis carried out showed that the saliency of the COVID-19 pandemic positively impacted state anxiety and, in turn, the personal need for structure. These results are in line with studies that showed how the COVID-19 pandemic is related to an increase in state anxiety (e.g., Maaravi and Heller, 2020; Rossi et al., 2020; Wong et al., 2020) due to the uncertainty that it has caused in the life of people (AbuRuz et al., 2019). Unclear and potentially threatening environmental situations can boost destructive or negative thoughts, negative feelings, and a physiological activation, leading to the inclination to imagine and reflect on the worst possible scenarios (state anxiety). In addition, as supported by many experts (Sollár, 2008), state anxiety is connected to an intensification of personal need for structure to decrease environmental insecurity and, as a consequence, mitigate anxiety symptoms (Thompson et al., 2013).

The analysis carried out showed also that state anxiety impacted the teachers’ tendency toward sustainability both directly and indirectly through the role of mediation of the personal need for structure. The direct relationship between state anxiety and the teachers’ tendency toward sustainability may be because, as already found by Di Maggio (2022), rising state anxiety levels connected to cognitive priming linked to COVID-19 pandemic may favor a greater negative tendency toward inclusion. The pandemic seems to favor the occurrence of a self-reinforcing spiral of negative emotions and thoughts that can negatively affect the propensity toward themes like inclusion and sustainability. These, in complicated times, can be seen as something difficult to implement and develop in new generations, thus not recognizing a positive and significant role in the construction of a more inclusive and sustainable society through their career activities.

It has also been found that state anxiety, through the mediational role of the personal need for structure, indirectly and negatively affected teachers’ tendency toward sustainability. On the contrary, it positively predicted the teachers’ tendency toward a conservative socio-economic vision. The mediational role of the personal need for structure in the relation between state anxiety and the propensity to consider the challenges of sustainable development toward a more conservative socioeconomic vision is in line with Karwowski et al. (2020) study. The research underlined how the need for structure, increased by a state of experienced anxiety, could lead individuals toward more conservative attitudes. In fact, for teachers, the propensity to consider the challenges of sustainable development in one’s career activities means questioning their knowledge, beliefs, and values as well as teaching procedures and the topics of their classes (Keleş, 2011, Keles, 2017). The other option would be to rely on the conservative economic approach: This means that they would “not venture into a new and unknown terrain.”

In conclusion, we can state that state anxiety and the personal need for structure, as shown by Karwowski et al. (2020), have a mediational role in impacting attitudes in pandemic times. In addition to this, this study showed how the need for structure may be considered a mediator exacerbating the relationship between anxiety and attitudes toward inclusion and sustainability.

This study has important theoretical and practical implications for teachers’ career development and career activities.

In terms of theoretical implications, in line with the career construction adaptation model (Savickas and Porfeli, 2012), this study emphasized the relevance to consider environmental features in career development. Specifically, this study adds to the career construction adaptation model that environmental features can impact people’s emotional state and consequently some positive tendency for the construction of a new and more sustainable society. Specifically, as in the case of COVID-19 pandemic, it is possible to hypothesize that experiencing negative external conditions, such as future economic crises or changes to the teachers’ employment contracts, may have negative consequences on the teachers’ emotional state (experiencing increased anxiety and consequently more need for structure) that can lead teachers to decrease their efforts in promoting sustainability and inclusion on their career activities. Having positive emotions and attitudes concerning inclusion and sustainability can help teachers to redefine their career roles by recognizing that their jobs have an active role in the building of sustainable future societies without relegating, as it often happens, their jobs only to a passive transmission of knowledge (Dunn et al., 2017). As recently emphasized by the career construction adaptation model (Guichard, 2018; Di Maggio et al., 2020; Nota et al., 2020), recognizing and reconceptualizing one’s work as useful and indispensable to building more inclusive and sustainable social contexts is a crucial aspect of career growth in current times. Furthermore, positive teachers’ emotions and attitudes concerning inclusion and sustainability can have an impact on their communication with their students and the development of their positive tendency toward these topics (Kalu and Ali, 2004; Borges, 2019; Nousheen et al., 2020).

From a practical perspective, the study suggested the relevance, of career counseling activities, to support workers, such as teachers, and help them consider the complexity of environmental aspects within the career development process and the impact that said aspects may have on the emotional state and attitudes and consequently, on career activities. Moreover, this study suggested the relevance of intervening at a political and systemic level to increase teachers’ positive propensity related to an inclusive and sustainable education in this complex period of uncertainty and intervening with training to support teachers to deal with complicated and unclear circumstances in which not only the education of students but also teachers’ wellbeing may be threatened.

In spite of these reassuring findings, we recognize some limitations of the study. First of all, we focused our attention on the environmental conditions/events than can affect psychological conditions leaving out personality traits. For this reason, we studied more “state” anxiety and “state” personal need of structure with respect to “trait” anxiety and “trait” personal need of structure. Nevertheless, keeping in mind that some personality traits may influence the perception of external and environmental events, in future studies, it may be useful to investigate the role of personality traits. Moreover, only self-reported data were used to analyze the dependent variables of the research. Furthermore, future research should involve both direct and indirect observations as other methodologies to decrease the influence of self-report bias. Another limitation concerns the much higher presence of female teachers in the sample. Although this gender-different distribution in the sample reflects an Italian phenomenon of occupational segregation in the school system (83% of female teachers; OECD., 2017), future studies should consider the role played by gender in the dimensions under investigation in this study. Moreover, only teachers from the northern part of Italy were involved and future research should involve teachers from different Italian regions to generalize the results achieved. Finally, although state anxiety symptoms tend to remain constant during both lockdown and reopened periods (Di Blasi et al., 2021), the results obtained in this study, related to the third wave of the COVID-19 pandemic, should not be cautiously generalized to the entire pandemic period.

## Data Availability Statement

The datasets presented in this article are not readily available because we prefer not to share data without first evaluating its use and intended purposes. Requests to access the datasets should be directed to ID, ilaria.dimaggio@unipd.it.

## Ethics Statement

The studies involving human participants were reviewed and approved by SIO – Italian Society for Vocational Guidance. The patients/participants provided their written informed consent to participate in this study.

## Author Contributions

ID was responsible for the design, writing, and data analysis phase. MG collaborated with ID on the theoretical background of the study and organized and supervised the assessment phase of the research. SS conducted the assessment phase. LN supervised the research work providing suggestions and guidance at all stages of the research work. All authors contributed to the article and approved the submitted version.

## Conflict of Interest

The authors declare that the research was conducted in the absence of any commercial or financial relationships that could be construed as a potential conflict of interest.

## Publisher’s Note

All claims expressed in this article are solely those of the authors and do not necessarily represent those of their affiliated organizations, or those of the publisher, the editors and the reviewers. Any product that may be evaluated in this article, or claim that may be made by its manufacturer, is not guaranteed or endorsed by the publisher.

## Appendix

**TABLE A1:** Italian and English versions of the stimuli used in the study.

Control condition	Un team internazionale di ricercatori ha annunciato un nuovo sviluppo che può cambiare il modo di lavorare e l’aspetto dei nostri portatili. La recente tendenza della tecnologia mobile, che si è facilmente diffusa e ha conquistato il mercato tecnologico del 2019, si è basata su un’idea semplice: permettere agli utenti di piegare i loro dispositivi. I telefoni cellulari pieghevoli hanno conquistato il cuore di molte persone, quindi non-sorprende che il passo successivo debba essere quello di creare computer portatili pieghevoli. Grazie alle soluzioni innovative, un portatile da 17 pollici con touch screen è diventato una realtà. I suoi progettisti sperano che il laptop superi i test iniziali con successo e che la produzione e la distribuzione in serie siano possibili entro la fine di giugno. La tecnologia è sicuramente un processo importante nella nostra società ed è in evoluzione Questo articolo di giornale di cosa tratta? (a) Nanotecnologia (b) Nuovo iPad (c) Computer portatili pieghevoli An international team of researchers has announced a new development that could change our work and the way our laptops look. The recent mobile technology trend, which has easily spread and taken over the 2019 tech market, was based on a simple idea: allowing users to fold their devices. Foldable cell phones have won the hearts of many people, so it’s no surprise that the next step should be to create foldable laptops. Thanks to innovative solutions, a 17-inch laptop with a touch screen has become a reality. Its designers hope that the computer will successfully pass initial tests and mass production and distribution will be possible by the end of June. Technology is an essential process in our society, and it is evolving What is about this journal article? a) Nanotechnology b) New iPad c) Foldable laptops

Experimental condition	La pandemia COVID 19 sembra non-arrestarsi più. Oggi come a Marzo, la diffusione del coronavirus sta seminando il panico in Italia e nel mondo. È iniziato tutto a Wuhan e ad oggi si contano ormai più di un milione di vittime. Anche in Italia il numero di positivi, così come quello delle vittime, continua a salire. Molti lo paragonano alla peste nera, che nell’Europa medievale ha portato alla morte di milioni di persone. Il Coronavirus diventerà una piaga moderna con un tasso di mortalità così inimmaginabile, che arriva fino al 40% dell’intera popolazione umana? Ma il Coronavirus non fa prigionieri e non ha pietà. Tra le vittime del virus ci sono persone di tutte le età, che svolgono lavori diversi, che abitano in luoghi diversi; ogni paese del mondo sta sperimentando la pandemia e non ci sono luoghi “immune” che possono essere considerate alla stregua di isole felici Questo articolo di giornale di cosa tratta? a) Movimento anti-vaccino b) Pandemia del Coronavirus c) Nuovo trattamento del cancro The COVID 19 pandemic seems never to stop. Today, as in March, the spread of the Coronavirus is spreading panic in Italy and around the world. It all started in Wuhan, and today there are more than one million victims. Even in Italy, the number of positives and the number of victims continue to rise. Many compare it to the Black Death, which in medieval Europe led to the death of millions of people. Will the Coronavirus become a modern plague with such an unimaginable mortality rate of up to 40% of the entire human population? But the Coronavirus takes no prisoners and shows no mercy. There are people of all ages among the virus victims, working in different jobs, living in other places; every country globally is experiencing the pandemic. There are no “immune” places that can be considered happy islands What is about this journal article? a) Anti-vaccine movement b) Coronavirus Pandemic c) New cancer treatment
